# A Comparative Study of Immunofluorescence on Formalin-Fixed, Paraffin-Embedded Versus Fresh Frozen Kidney Biopsy

**DOI:** 10.7759/cureus.40978

**Published:** 2023-06-26

**Authors:** Nipan Das, Rennie O Lakadong, Biswajit Dey, Vandana Raphael

**Affiliations:** 1 Pathology, North Eastern Indira Gandhi Regional Institute of Health and Medical Sciences (NEIGRIHMS), Shillong, IND; 2 Allied Health Sciences, Martin Luther Christian University, Shillong, IND

**Keywords:** kidney disease, proteinase, antigen retrieval, formalin-fixed paraffin-embedded sections, direct immunofluorescence

## Abstract

Background

Immunofluorescence techniques done on formalin-fixed, paraffin-embedded tissue can serve as salvage techniques in cases where immunofluorescence on the frozen section may not be adequate or available. The present study was undertaken to assess the diagnostic utility of paraffin immunofluorescence by proteinase K digestion on renal biopsy compared to fresh frozen immunofluorescence.

Methodology

The paraffin immunofluorescence by proteinase K digestion of paraffin-embedded renal biopsy (IF-FFPE) was standardized and compared with the immunofluorescence on fresh frozen tissue (IF-Frozen). A total of 50 cases of the native renal biopsy were included in the study, and their intensity for fluorescein isothiocyanate-labeled IgA, IgG, IgM, C3, kappa, and lambda was compared.

Results

A total of 50 cases of the native renal biopsy were included in the study, and their intensity for fluorescein isothiocyanate-labeled antibodies of IgA, IgG, IgM, C3, kappa, and lambda was compared. The difference of 2+ intensity of antibodies between IF-FFPE and IF-Frozen was noted mainly in lupus nephritis (15%), followed by IgA nephropathy (10%) and membranoproliferative glomerulonephritis (7%). IF-FFPE showed a sensitivity of 90.3%, 91.8%, 82.7%, 81.1%, 92.1%, and 94.6% for IgA, IgG, IgM, C3, kappa, and lambda, respectively, whereas specificity was 100% for IgA, IgG, C3, kappa, and lambda and 95.2% for IgM.

Conclusions

Immunofluorescence techniques done on formalin-fixed, paraffin-embedded tissue can serve as salvage techniques in kidney biopsies.

## Introduction

Direct immunofluorescence on fresh frozen tissue has long been the gold standard for the detection of immune complexes and complements in renal immunopathological diagnosis. However, there are some disadvantages to immunofluorescence on fresh frozen tissue in clinical practice. The disadvantages include a thick frozen section, or the antigens may appear to have a diffuse distribution, leading to analytical difficulties [1]. Moreover, scant frozen tissue may decrease diagnostic accuracy. Lastly, the frozen section cannot be retrospectively analyzed for re-evaluation. In these scenarios, an alternative technique done on formalin-fixed, paraffin-embedded tissue may serve as a salvage technique. Studies have revealed that immunofluorescence techniques on formalin-fixed, paraffin-embedded tissue give comparable results to those obtained on frozen sections for most pathogenic immunoglobulins and complements [1]. However, formalin fixation of renal tissues causes a masking effect of antigens due to extensive protein cross-linking which blocks the accessibility of fluorescein isothiocyanate (FITC)-conjugated antibodies to interact with the antigens [2-4].

To overcome the problem of masking of antigens in formalin-fixed, paraffin-embedded tissues, various antigen retrieval methods are used. Antigen retrieval methods such as proteinase K, pronase, trypsin, and dual microwave heating have been used for immunofluorescence techniques on formalin-fixed, paraffin-embedded kidney biopsies [3,4]. Even though this method has been documented in the literature using various enzymes, it is still not commonly used in laboratories that process renal biopsies. In this study, proteinase K was utilized for antigen retrieval. Proteinase K is an enzyme that aids in the breakdown of protein cross-linkages created during formalin fixation, exposing the antigenic immune complexes to FITC-labeled antibody staining [3,4].

The present study was undertaken to assess the diagnostic utility of paraffin immunofluorescence by proteinase K digestion on renal biopsy compared to fresh frozen immunofluorescence.

## Materials and methods

All archived formalin-fixed, paraffin-embedded blocks for routine hematoxylin and eosin (H&E)-stained slides for which corresponding direct immunofluorescence on a frozen section was available were collected. To perform immunofluorescence on formalin-fixed, paraffin-embedded tissue (IF-FFPE), proteinase K (Sigma-Aldrich, USA, Cat. P2308) was applied to the slides prepared from the same blocks from which H&E-stained slides were created for the histopathological interpretation of kidney diseases.

Proteinase K method

In the proteinase K method of antigen retrieval, poly-L-lysine-coated slides were taken with 3 µm sections. Deparaffinization was done, followed by rehydration, and kept in Tris buffer at a pH of 9.0. The unmasking of antigens was done by adding proteinase K. Subsequently, the slides were incubated in a humidified wet chamber. After incubation, fluorescence-conjugated polyclonal antibodies, which included IgA, IgG, IgM, C3, kappa, and lambda (Dako, Carpinteria, CA, USA), were added. Finally, slides were rinsed in phosphate-buffered saline and mounted in aqueous phosphate buffer glycerol. The results were compared to those of direct immunofluorescence on frozen sections.

Frozen section method

In the frozen section method (IF-Frozen), poly-L-lysine-coated slides with 3-4 µm sections were taken. The 3-4 µm sections were cut in a cryostat. The sections were first dried and washed in phosphate-buffered saline three times at a pH of 7.4. Fluorescence-conjugated antibodies were added and incubated at 37°C. Again, the slides were washed in phosphate-buffered saline three times. The slides were then mounted with glycerine.

Scoring of immunofluorescence

The scoring was done at 400× magnification, and the interpretation was done as follows: no staining (0), mild staining (1+), moderate staining (2+), moderate-to-high staining (3+), and high staining (4+). The intensity of FITC-labeled antibodies of IgA, IgG, IgM, C3, kappa, and lambda was compared in terms of average intensity and intensity difference between IF-FFPE and IF-Frozen. The sensitivity and specificity of the IF-FFPE were compared to that of the gold standard method of the IF-Frozen technique. All the IF-FFPE were interpreted by a single nephropathologist who was blinded to the findings of the IF-Frozen.

## Results

A total of 50 cases of native renal biopsies were included in the study. There were 14 cases of membranoproliferative glomerulonephritis (MPGN), 13 cases of lupus nephritis, 10 cases of IgA nephropathy, five cases of minimal change disease (MCD), five cases of post-infectious glomerulonephritis (PIGN), and three cases of focal segmental glomerulosclerosis (FSGS). In these 50 cases, the intensity of FITC-labeled antibodies of IgA, IgG, IgM, C3, kappa, and lambda was compared between IF-FFPE and IF-Frozen (Table 1).

**Table 1:** The average intensity with intensity difference by paraffin immunofluorescence method compared with fresh frozen immunofluorescence. MPGN: membranoproliferative glomerulonephritis; MCD: minimal change disease; PIGN: post-infectious glomerulonephritis; FSGS: focal segmental glomerulosclerosis; IF-F: immunofluorescence on fresh frozen tissue; IF-FFPE: immunofluorescence on formalin-fixed, paraffin-embedded tissue

		IgA	IgA	IgG	IgG	IgM	IgM	C3	C3	Kappa	Kappa	Lambda	Lambda
Diagnosis	Cases (n = 50)	IF-F	IF-FFPE	IF-F	IF-FFPE	IF-F	IF- FFPE	IF-F	IF-FFPE	IF-F	IF-FFPE	IF-F	IF-FFPE
MPGN	14	0.86	0.57 (1+)	1.64	1.43 (1+)	0.86	0.78 (1+)	0.93	0.57 (1+)	1.28	1.21 (1+)	1.28	1.14 (1+)
Lupus nephritis	13	2.46	2.15 (1+)	2.92	2.85 (1+)	2.08	1.78 (1+)	2.15	1.61 (1+)	2.61	2.61 (equal)	2.61	2.46 (1+)
IgA nephropathy	10	2.6	2.2 (1+)	0.8	0.8 (equal)	0.3	0.45 (equal/ better )	1.5	1.3 (1+)	1.5	1.4 (1+)	1.4	1.4 (equal)
MCD	5	0	0	0	0	0	0	0	0	0	0	0	0
PIGN	5	0.6	0.6 (equal)	2.4	2.6 (equal/better)	0.6	0.6 (equal)	1.6	1.6 (equal)	1.8	1.6 (1+)	2	1.8 (1+)
FSGS	3	0.66	0.33 (2+)	2.33	2 (1+)	0.66	0.66 (equal)	1.33	1 (1+)	2.33	2.33 (equal)	2.33	2.33 (equal)

**Figure 1:**
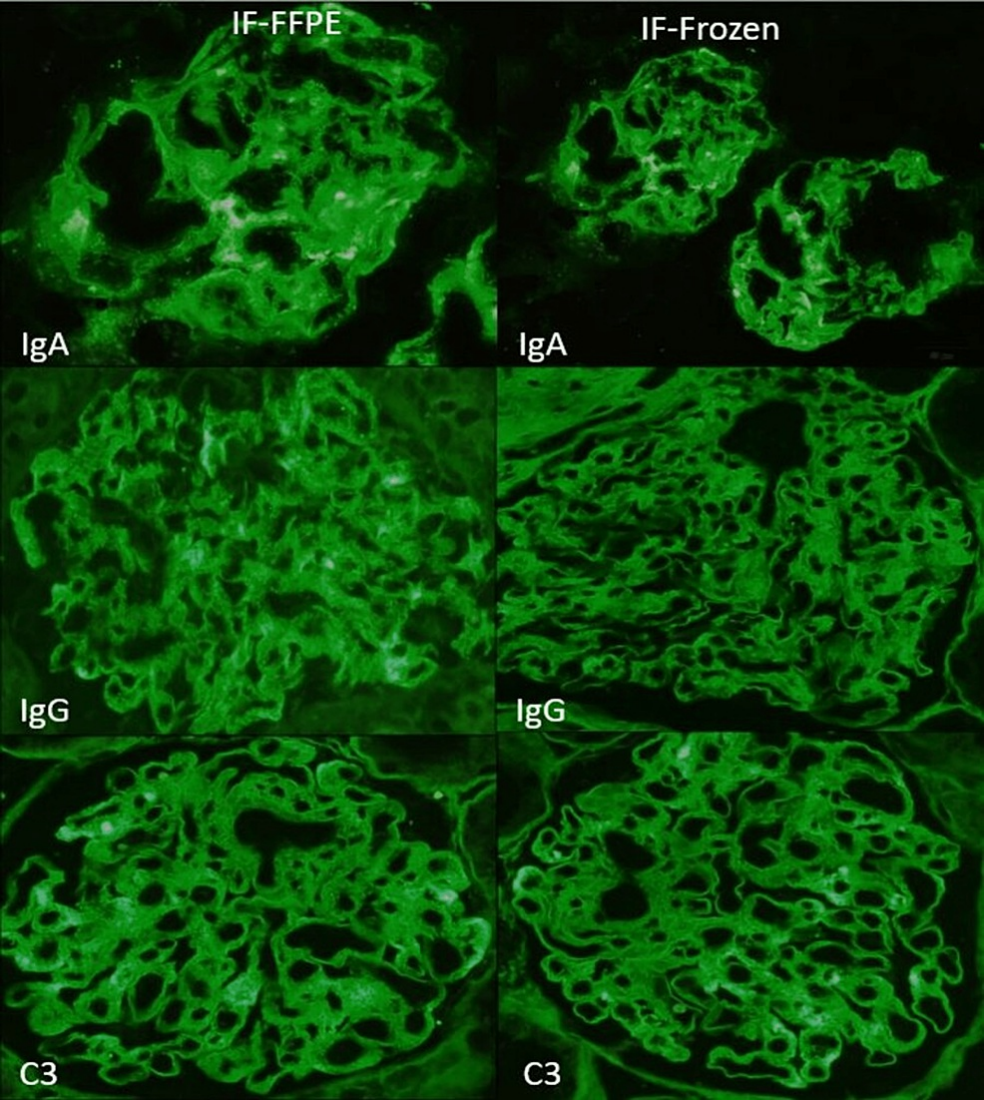
Photomicrographs showing a comparison of immunofluorescence on fresh frozen tissue (FITC-tagged antibodies using IgA in 10×, IgG in 20×, and C3 in 20×) and formalin-fixed, paraffin-embedded section (FITC-tagged antibodies using IgA in 20×, IgG in 20×, and C3 in 20×) of a case of IgA nephropathy. IF-FFPE: immunofluorescence in formalin-fixed, paraffin-embedded; IF-Frozen: immunofluorescence in frozen section; FITC: fluorescein isothiocyanate

The equal intensity of FITC-labelled antibodies was observed in 86% of MPGN, 46% of lupus nephritis, 60% of IgA nephropathy, 80% of PIGN, and 100% of FSGS cases (Figure 1). The difference in 2+ intensity of immunoglobulins or complement between IF-FFPE and IF-Frozen was noted mainly in lupus nephritis (15%), followed by IgA nephropathy (10%) and MPGN (7%) (Table 2).

**Table 2:** Immunofluorescence on fresh frozen tissue versus formalin-fixed, paraffin-embedded tissue. MPGN: membranoproliferative glomerulonephritis; MCD: minimal change disease; PIGN: post-infectious glomerulonephritis; FSGS: focal segmental glomerulosclerosis; IF-F: immunofluorescence on fresh frozen tissue; IF-FFPE: immunofluorescence on formalin-fixed, paraffin-embedded tissue

Diagnosis	Equal intensity of immunoglobulin/complement between IF-F and IF-FFPE	Difference of 1+ in the intensity of immunoglobulin/complement between IF-F and IF-FFPE	Difference of 2+ in the intensity of immunoglobulin/complement between IF-F and IF-FFPE
MPGN (n = 14)	12 (86%)	01 (7%)	01 (7%)
Lupus nephritis (n = 13)	06 (46%)	05 (39%)	02 (15%)
IgA nephropathy (n = 10)	06 (60%)	03 (30%)	01 (10%)
PIGN (n = 5)	04 (80%)	01 (20%)	-
MCD (n = 5)	05 (100%)	-	-
FSGS (n = 3)	03 (100%)	-	-
n = 50	n = 36 (72%)	n = 10 (20%)	n = 04 (8%)

Among the immunoglobulins and complements, C3, followed by IgA and IgM, showed a difference of 2+ intensity in these cases (Table 3).

**Table 3:** Cases with a difference of 2+ in the intensity of antibodies between IF-F and IF-FFPE. MPGN: membranoproliferative glomerulonephritis; IF-F: immunofluorescence on fresh frozen tissue; IF-FFPE: immunofluorescence on formalin-fixed, paraffin-embedded tissue

Case: Diseases with a difference of 2+ intensity	Antibodies
MPGN (n = 01)	IgA and C3
Lupus nephritis (n = 02)	IgM and C3; only C3
IgA Nephropathy (n = 01)	Only IgA

The specificity for IF-FFPE was 100% for IgA, IgG, C3, kappa, and lambda. IgM showed a specificity of 95.2%. The sensitivity was the highest for lambda (94.6%), followed by kappa (92.1%). The sensitivity for IF-FFPE was the lowest for C3 (81.1%), followed by IgM (82.7%) (Table 4).

**Table 4:** Cases with a difference of 2+ in the intensity of antibodies between IF-F and IF-FFPE. IF-F: immunofluorescence on fresh frozen tissue; IF-FFPE: immunofluorescence on formalin-fixed, paraffin-embedded tissue

IF- F/IF-FFPE	a (+/+)	b (-/+)	c (+/-)	d (-/-)	Number of cases	Sensitivity a/(a+c)	Specificity d/(d+b)
Ig A	28	0	3	19	50	90.3%	100%
IgG	34	0	3	13	50	91.8%	100%
IgM	24	1	5	20	50	82.7%	95.2%
C3	30	0	7	13	50	81.1%	100%
Kappa	35	0	3	12	50	92.1%	100%
Lambda	35	0	2	13	50	94.6%	100%

## Discussion

Immunofluorescence on formalin-fixed, paraffin-embedded tissue requires unmasking of antigens as the masking effect of formalin fixation, which occurs from protein cross-linking, prevents FITC-conjugated antibodies from interacting with the antigens. Several techniques, including enzyme digestion and heat treatment, have been tried to reveal the antigen. For antigen retrieval, the enzymes trypsin, pepsin, protease VII, pronase, protease XXIV, and proteinase K have been utilized for varying amounts of time, temperature, and concentration [5].

In a study by Solanki et al., 92% of cases were diagnosed based on immunofluorescence done on formalin-fixed, paraffin-embedded tissue by proteinase K antigen retrieval on kidney biopsies compared to immunofluorescence done on frozen sections [5]. A similar methodology was also used by Wagrowska-Danilewicz et al., Nada et al., Singh et al., and Mohammadzadeh et al. [2,6-8]. They found good concordant results in kidney biopsy samples and concluded that it was a valuable salvage technique for renal biopsies. In this study, we evaluated the immunofluorescence on formalin-fixed, paraffin-embedded tissue by proteinase K antigen retrieval on kidney biopsies and compared the findings with immunofluorescence done on fresh frozen sections of kidney biopsies.

Solanki et al. found equal intensity or a difference of 1+ for IgG, IgM, C1q, kappa, and lambda light chains in most of the cases. In three cases, one from each of lupus nephritis, immune complex glomerulonephritis, and membranous nephropathy, IgG showed a difference of 2+ intensity on IF-FFPE in comparison to IF-Frozen [5]. Mohammadzadeh et al. also found equal intensity or a difference of 1+ for IgG, IgM, and C3 in most of the cases except in one case of IgA nephropathy with a difference of 2+ intensity for IgA [8]. In this study, a difference of 2+ intensity between IF-FFPE and IF-Frozen was noted in two cases of lupus nephritis (IgM and C3), one case of IgA nephropathy (IgA), and one case of MPGN (IgA and C3). These findings suggest that most of the antibodies in kidney biopsies show equal intensity or a minor difference of 1+ on IF-FFPE in comparison to IF-Frozen. Immunoglobulins and complements can be picked up by proteinase K digestion in conditions where immunological deposits are moderate to profuse. However, it is challenging to diagnose renal pathologies in the early stage with mild deposits because IF-FFPE is less sensitive than IF-Frozen [6].

Renal pathologists should be cautious in interpreting IgG, IgA, and C3, especially linear IgG in anti-glomerular basement membrane disease, C3 in C3 glomerulonephritis, and IgA in IgA nephropathy [1,3]. In this study, a difference of 2+ intensity was noted in antibodies of C3 in two cases of lupus nephritis and IgA in one case of IgA nephropathy. IgA nephropathy, which is characterized by dominant or co-dominant mesangial IgA immune deposits, with weak IgA may show false-negative staining for IgA on IF-FFPE [3,9]. There were no cases of C3 glomerulonephritis and anti-glomerular basement membrane disease in the present study cohort.

In the present research, the sensitivity and specificity of the antibodies against IF-FFPE were calculated. IF-FFPE showed a sensitivity of 90.3%, 91.8%, 82.7%, 81.1%, 92.1%, and 94.6% for IgA, IgG, IgM, C3, kappa, and lambda, respectively, whereas specificity was 100% for IgA, IgG, C3, kappa, and lambda and 95.2% for IgM. Mohammadzadeh et al. also calculated the sensitivity and specificity of antibodies on IF-FFPE compared to IF-Frozen [8]. They found a sensitivity of 93.1%, 76.9%, 63.6%, and 33.3%, and a specificity of 100%, 97.3%, 95%, and 100% for IgG, IgA, IgM, and C3, respectively [8]. Similarly, Alwahaibi et al. also showed that IgA, IgG, and IgM had a specificity of 84.8%, 69.2%, and 66.7%, respectively, whereas the sensitivity of IgA, IgG, and IgM was 61.8%, 74.2%, and 64.2%, respectively [10]. These findings suggest that IF-FFPE has high specificity. Among the antibodies, the sensitivity of IgM and C3 might be slightly lower than their other counterparts.

Despite its lower sensitivity, IF-FFPE has found its primary utility in two renal entities, which are membranous-like glomerulopathy with masked IgG kappa deposits and MPGN with masked monotypic immunoglobulin deposits [1,11,12]. These entities are often misdiagnosed as C3 glomerulonephritis on IF-Frozen and thus require IF-FFPE for unmasking other immunoglobulins by enzyme digestion [1,11,12]. No such entities were encountered in the present study cohort.

Limitations of the study were a relatively smaller sample size and the employment of one method of antigen retrieval in IF-FFPE. Future studies with larger sample sizes encompassing various other renal pathologies and comparison of different methods of antigen retrieval for IF-FFPE are recommended. Moreover, studies employing non-formalin-based fixatives such as the Bouin solution will be of immense interest. Other limitations of IF-FFPE such as non-specific background staining, autofluorescence of intraluminal serum in glomerular capillaries, variable staining among the glomeruli, and lesser appreciation of the granular nature of deposits may be overcome with awareness and practice among renal pathologists.

## Conclusions

In circumstances when immunofluorescence on the frozen section may not be adequate or available, immunofluorescence techniques performed on formalin-fixed, paraffin-embedded tissue can be a useful approach. The proteinase K digestion technique for immunofluorescence on formalin-fixed, paraffin-embedded renal tissue has good concordance in terms of intensity compared to immunofluorescence on fresh frozen kidney biopsies. The authors suggest immunofluorescence done on formalin-fixed, paraffin-embedded tissue should not replace standard immunofluorescence with frozen sections when assessing renal biopsies and should be viewed as a salvage approach to increase the sensitivity and specificity of detecting immunoglobulins and complements in various kidney pathologies.
